# CYLD Limits Neutrophil-Driven Psoriatic Inflammation

**DOI:** 10.1007/s10753-026-02452-3

**Published:** 2026-01-20

**Authors:** Zhenzong Fa, Zeping Huang, Yi Shang, Yang Yang, Qun Xie, Runping Yang

**Affiliations:** 1 Department of Dermatology, the Sixth Medical Center, Chinese PLA General Hospital, Beijing, 100048 China; 2 Outpatient Department, the Sixth Medical Center, Chinese PLA General Hospital, Beijing, 100048 China; 3 Outpatient Department, Chinese PLA General Hospital, Beijing, 100048 China; 4 Department of Anesthesiology, the Fourth Medical Center, Chinese PLA General Hospital, Beijing, 100048 China

**Keywords:** Psoriasis, CYLD, Neutrophil, Neutrophil extracellular traps.

## Abstract

Despite the well-recognized role of the deubiquitinase CYLD in the pathogenesis of tumors and certain inflammatory diseases, its specific function and regulatory mechanisms in psoriasis remain unclear. Thus, we first analyzed CYLD expression differences between psoriatic patients and healthy controls using skin samples from the GEO database and validated its expression dynamics in an imiquimod (IMQ)-induced mouse model. Then, we systematically evaluated the effects of CYLD deficiency on psoriasiform inflammation through histopathology, immunohistochemistry, RNA sequencing, and immunofluorescence analyses using Cyld knockout (*Cyld*^*−/−*^) mice, and employed bioinformatics approaches including CIBERSORT and Weighted Gene Co-expression Network Analysis (WGCNA) to further explore the associations between CYLD and neutrophil-related pathways and genes. The results showed that CYLD expression was significantly upregulated in lesional skin of psoriasis patients; *Cyld*^*−/−*^ mice displayed more severe psoriasiform symptoms (enhanced epidermal thickening, increased neutrophil infiltration, significantly augmented formation of neutrophil extracellular traps [NETs]); CYLD deficiency led to excessive activation of the NF-κB signaling pathway and upregulated expression of various pro-inflammatory cytokines and chemokines. Bioinformatics analyses confirmed CYLD was closely associated with pathways related to neutrophil migration and activation. These findings lead to the conclusion that CYLD plays a crucial negative regulatory role in psoriasis by inhibiting NF-κB-mediated neutrophil activation and NETs formation, so targeted activation of CYLD may represent a promising novel therapeutic strategy for psoriasis.

**Supplementary Information:**

The online version contains supplementary material available at 10.1007/s10753-026-02452-3.

## Introduction

Psoriasis is a prevalent chronic inflammatory skin disorder characterized by a complex pathogenesis involving various immune cell interactions. It is estimated that globally, psoriasis affects approximately 2–3% of the population, with prevalence varying across different regions and demographics [1]. The condition is marked by dysregulated immune responses, particularly involving the activation of immune cells such as T cells and neutrophils, as well as the production of various pro-inflammatory cytokines [2]. Central to the pathophysiology of psoriasis is the IL-23/IL-17 axis, identified as a critical signaling pathway driving the inflammatory processes in the disease [3]. The interplay between innate and adaptive immune responses is also significant, with neutrophils emerging as key players in the inflammatory milieu of psoriatic lesions [4–8].

Neutrophils, the most abundant type of white blood cells, are frequently found in high numbers within psoriatic skin lesions and are believed to contribute significantly to the disease’s pathogenesis [6]. They produce a variety of cytokines and chemokines that promote inflammation, including IL-17 and IL-36, which further exacerbate the inflammatory response. Additionally, neutrophils can form extracellular traps (NETs) that entrap pathogens but may also contribute to tissue damage and chronic inflammation in psoriasis [8–10]. The mechanisms underlying neutrophil activation involve several signaling pathways, including the NF-κB pathway, which is essential for the expression of pro-inflammatory genes [11]. However, these mechanisms are not yet fully understood.

CYLD is a ubiquitin hydrolase that belongs to the ubiquitin-specific protease (USP) family of deubiquitinating enzymes (DUBs), which play critical roles in immunity, cell homeostasis, and disease pathogenesis [12, 13]. Studies across various model systems have revealed that these processes are mediated through CYLD’s regulation of cellular pathways, including the NF-κB [14], Wnt [15], and TGF-β pathways [16]. However, the role of CYLD in regulating psoriatic inflammation remains unclear. In this article, we found that CYLD depletion leads to exacerbated psoriatic inflammation, and this effect may be related to neutrophil infiltration and activation.

## Results

### Psoriasis Pathogenesis is Associated with CYLD Expression

**Fig. 1:**
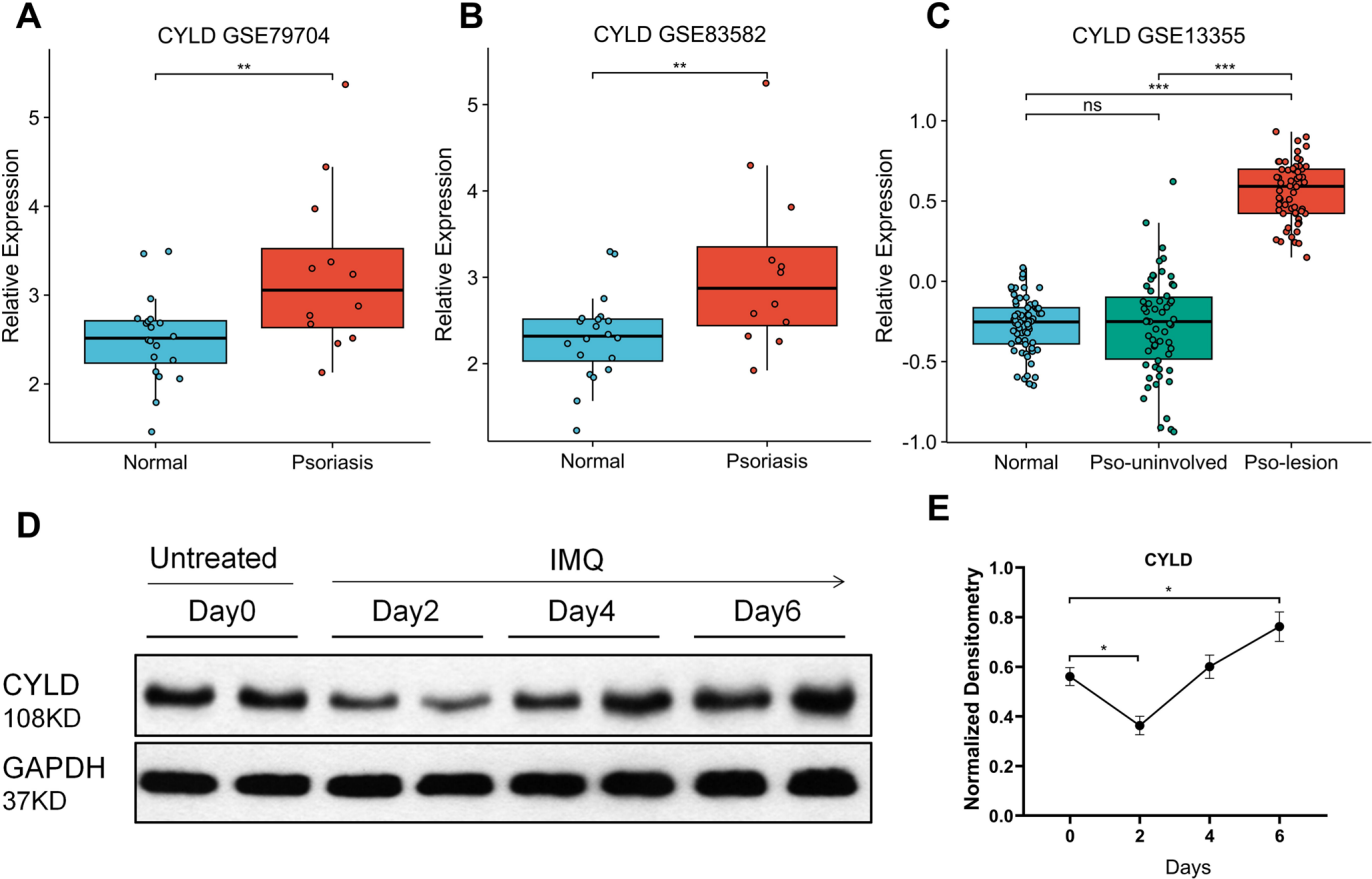
Psoriasis is associated with changes in CYLD expression. **A**-**C** CYLD levels were analyzed in human skin samples from healthy (normal) individuals and psoriasis patients (Psoriasis). Data were extracted from publicly available data sets GSE79704 (A), GSE83582 (B), and GSE13355 (C), respectively. “Pso - uninvolved” refers to samples of healthy skin from psoriasis patients, while “Pso - lesion” denotes samples of psoriasis lesions. **D**, **E** The expression of CYLD in lesional skin was measured on days 0, 2, 4, and 6 in an IMQ - induced murine model. The Figure 1(D) shows two representative samples from two independent experiments; The Figure 1(E) shows data of four independent experiments, with four mice in each group. *p<0.05,**p<0.01,***p<0.001, ns=no significant difference

CYLD has been implicated in the development of various inflammatory diseases [12, 13, 17]. Moreover, recent studies indicate that several mutations of CYLD could influence the pathogenesis of psoriasis [18]. To investigate this further, we first analyzed CYLD expression levels in skin samples from healthy individuals and psoriatic patients using publicly available datasets from the Gene Expression Omnibus (GEO). Three independent datasets were analyzed: GSE79704 [19, 20] (20 healthy individuals and 12 psoriasis patients), GSE83582 [19] (20 healthy vs. 12 psoriatic samples), and GSE13355 [21–23] (64 healthy vs. 58 psoriatic samples). Across all datasets, CYLD expression was significantly up-regulated in psoriatic skin compared with healthy controls (Fig. 1A-C). Notably, CYLD expression in psoriatic lesional skin was also significantly up-regulated compared to non-lesional skin from psoriasis patients. To determine whether this observation is conserved in psoriatic animal models, we analyzed CYLD expression in an imiquimod(IMQ)-induced psoriasis-like mouse model. Interestingly, CYLD expression initially decreased during the first two days following IMQ application, but then increased continuously from day 2 to day 6, reaching approximately 1.5-fold the baseline level (Day 0) by day 6. Collectively, these findings suggest a strong association between CYLD and the pathogenesis of psoriasis.

### CYLD Deficiency Exacerbates Psoriatic Phenotypes and Enhances Inflammatory Cell Infiltration

**Fig. 2:**
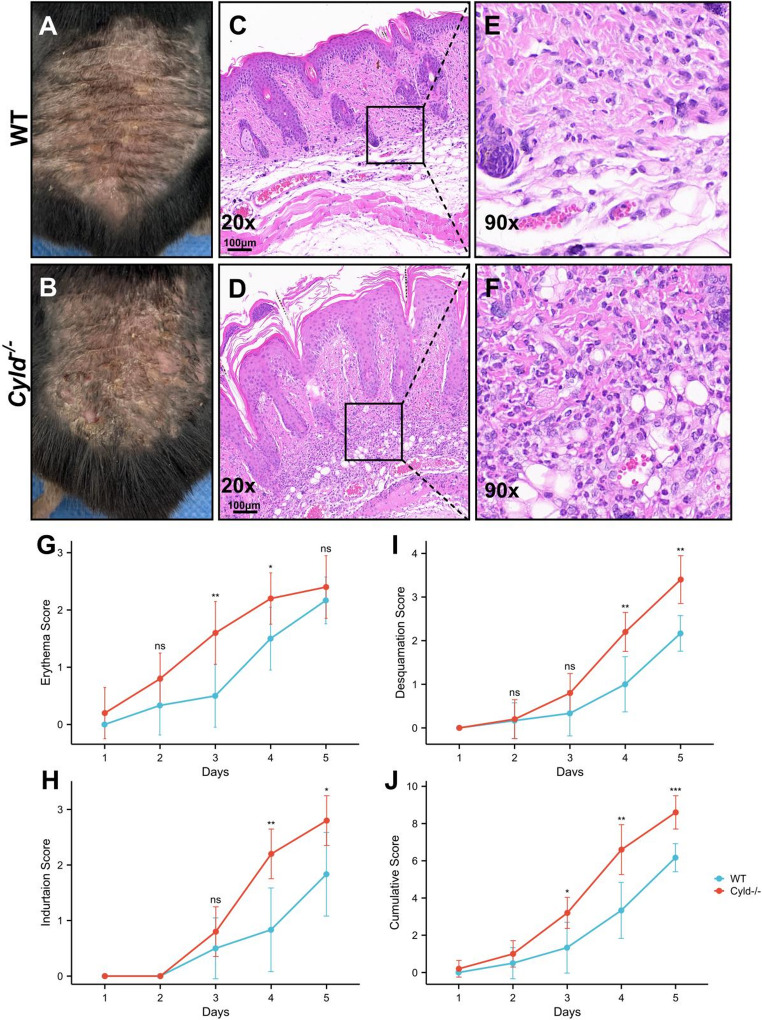
*Cyld* depletion leads to deterioration of IMQ-induced psoriasis with increased neutrophil infiltration. **A**, **B** Representative images of dorsal skin from WT mice (A) and *cyld* ^*−/−*^ mice (B) after treatment with IMQ for 5 days. Notably, *cyld* ^*−/−*^ mice showed more prominent erythema and scaly lesions. **C**-**F** H&E staining showed typical psoriasiform changes in both the WT (C) and *cyld* ^*−/−*^ groups (D). *Cyld* depletion resulted in a thicker spinous cell layer (C, D) and more obvious dermal inflammatory infiltration (E, F). **G**, **H** Erythema, induration, and desquamation scores of dorsal lesions were evaluated daily from Day 1 in both wild-type (WT) and *cyld* ^*−/−*^ mice. The cumulative score was calculated as the sum of erythema, induration, and desquamation scores at each indicated time point (*p < 0.05, **p < 0.01, ***p < 0.001, ns = no significant difference). This experiment was repeated three times, with five mice in each group

To investigate the function of CYLD in psoriasis development, we employed *Cyld* global knockout (*Cyld*^*−/−*^) mice and their wild-type (WT) littermates. Prior to treatment, *Cyld*^*−/−*^ mice show no significant differences from WT mice in skin gross morphology and histopathology. Mice were treated with 62.5 mg IMQ per mouse per day, and psoriatic characteristics (erythema, desquamation, and induration) were evaluated. Compared with WT controls, *Cyld*^*−/−*^ mice exhibited more severe psoriasiform features including marked hyperemia and extensive crusting with desquamation (Fig. 2A, B). These macroscopic changes were confirmed by significantly higher individual and cumulative clinical scores (Fig. 2G-J). Consistently, pathological examination revealed that CYLD deficiency led to more pronounced acanthosis, elongation of epidermal ridges, neutrophilic microabscesses, and robust inflammatory cell infiltration in the superficial and mid-dermis (Fig. 2C, D). High-power magnification identified neutrophils and lymphocytes as the predominant infiltrating cell types (Fig. 2E, F). Collectively, these data indicate that CYLD deficiency exacerbates IMQ-induced psoriasiform phenotypes and promotes inflammatory cell infiltration.

### CYLD Depletion Promotes Ki67 Expression and Neutrophil Infiltration in IMQ-induced Lesions

**Fig. 3:**
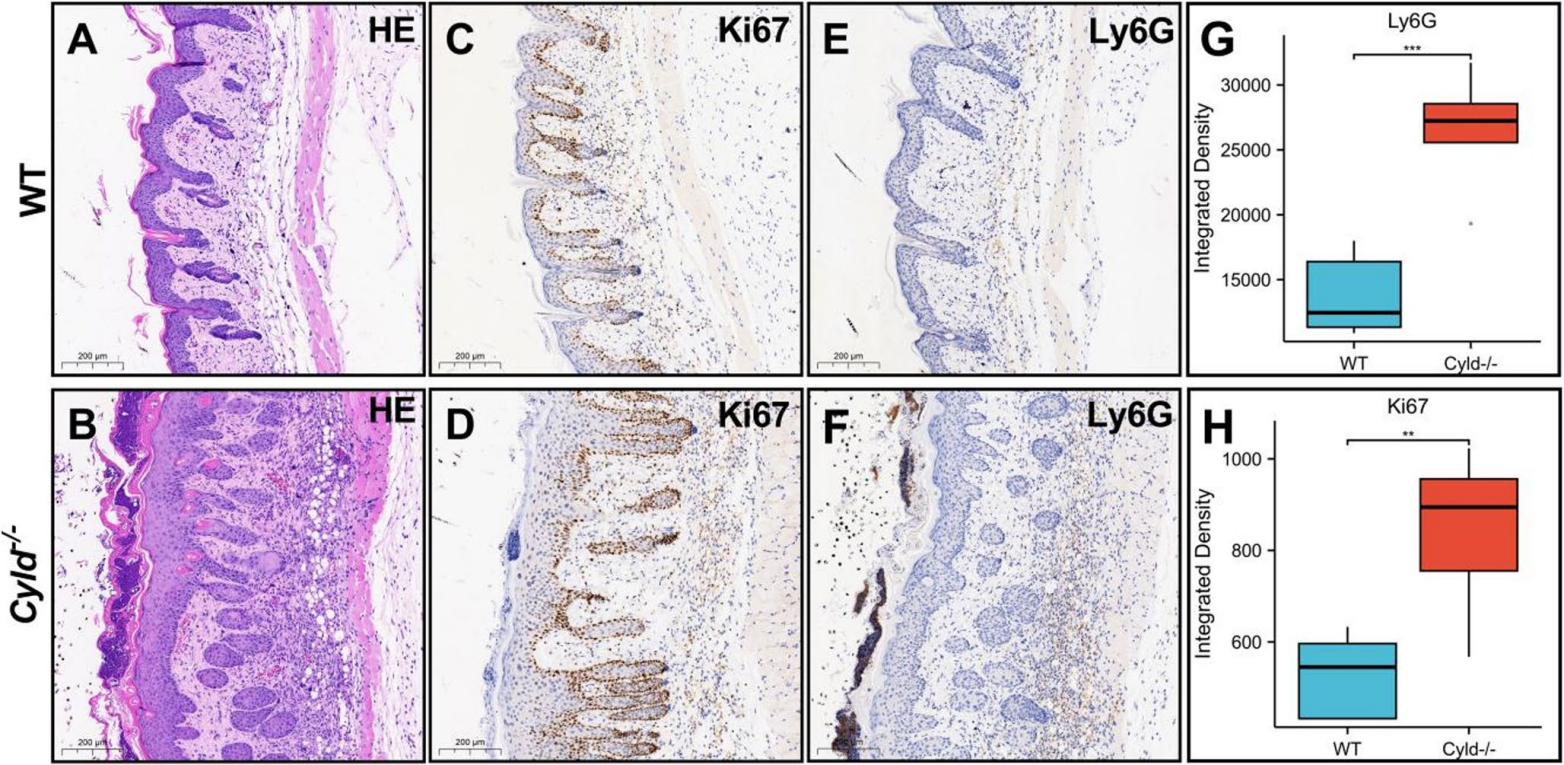
*Cyld* depletion promotes *Ki67* expression and neutrophil infiltration in IMQ-induced psoriasis lesions. **A**, **B** HE staining visualizes the tissue background for immunohistochemical analysis of Ki67 (**C**, **D**) and Ly6G (**E**, **F**). **G**, **H** Quantitation of Ly6G and Ki67 positive signals analyzed by NIH Image J software. **p < 0.01, ***p < 0.001. This experiment was repeated three times, with three mice in each group

We next performed immunohistochemical staining to assess keratinocyte proliferation and inflammatory infiltration. In WT mice, Ki67-positive cells were primarily located in the basal layer, with a small number in the superficial dermis. In contrast, *Cyld*^*−/−*^ mice displayed a significant increase in Ki67-positive cells within the basal layer, and positive cells were also evident throughout all layers of the epidermis (Fig. 3C, D). Ly6G-positive neutrophils were mainly distributed in the dermis of WT mice and were barely detectable in the epidermis and stratum corneum (Fig. 3E). CYLD depletion resulted in a significant increase in Ly6G-positive cell counts (Fig. 3F, G), with numerous positive cells accumulating in the stratum corneum (Fig. 3F), consistent with the ‘Munro-like’ microabscesses observed by HE staining (Fig. 1C, D). These findings suggest that CYLD depletion promotes keratinocyte hyperproliferation and enhances neutrophil infiltration in IMQ-induced psoriatic lesions.

### CYLD Deficiency Promotes Neutrophil Infiltration and Activation in the IMQ Model

**Fig. 4:**
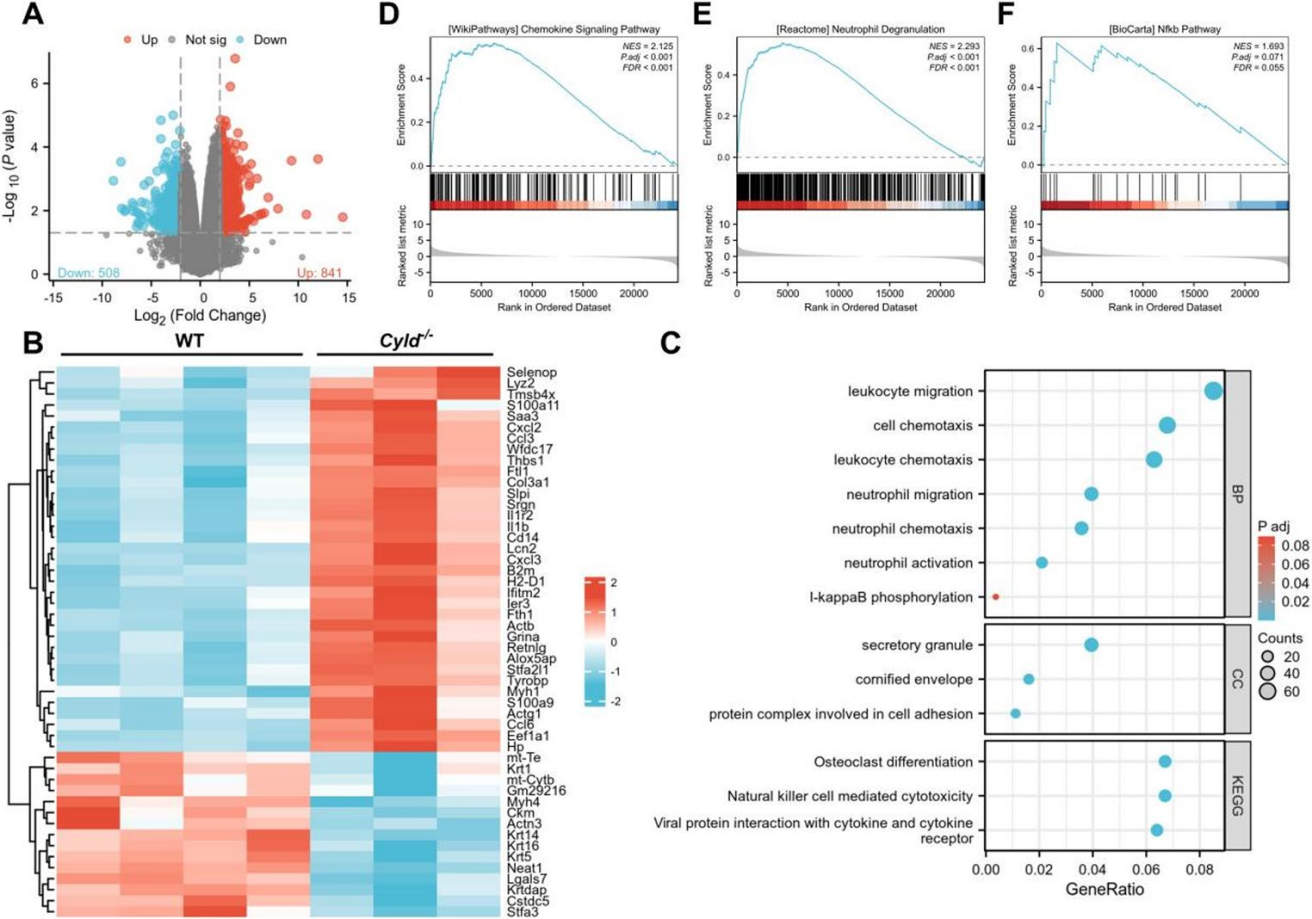
RNA-seq analysis suggests that *Cyld* deficiency may promote neutrophil infiltration and activation in the IMQ model. **A** Volcano plot of differentially expressed genes (DEGs). Red dots represent significantly upregulated genes (*n* = 841), blue dots represent significantly downregulated genes (*n* = 501), and gray dots represent non-significant genes. **B** Heatmap depicting the 50 most significant differentially expressed genes. Blue indicates higher expression in WT mice, while red indicates higher expression in *Cyld*^*−/−*^ mice. Genes are clustered by their expression divergence patterns. **C** KEGG pathway and GO enrichment analyses. Results show prominent enrichment of pathways related to neutrophil migration, chemotaxis, and activation. **D**-**F** Gene set enrichment analysis (GSEA) of neutrophil infiltration and activation pathways.This experiment was repeated twice, with three mice in WT group and four mice in *cyld* ^*−/−*^ group

To further investigate the function of CYLD in regulating psoriasis development, we performed RNA sequencing analysis (RNA-seq) to compare gene expression alternations between IMQ-treated *Cyld*^*−/−*^ mice and WT mice (time point day 5). A volcano plot depicting changes in differentially expressed genes (DEGs) of two groups is shown in Fig. 4A. Compared with the WT group, 1349 genes were significantly altered, including 508 down-regulated and 841 up-regulated in the *Cyld*^*−/−*^ group (Log|fold change[FC]|≥2, *P* < 0.05). The top 50 DEGs were shown in a heatmap (Fig. 4B), revealing that a large part of up-regulated genes in *Cyld*^*−/−*^ group are associated with inflammatory pathways, including many pro-inflammatory cytokines and chemokines. Notably, genes such as *S100a10*, *Cxcl2*, and *Ccl3* were markedly increased. Further analysis via KEGG pathway and GO enrichment confirmed that pathways associated with inflammation, such as the leukocyte migration, leukocyte chemotaxis, neutrophils migration, neutrophils activation and NF-κB signaling, were significantly enriched in the *Cyld*^*−/−*^ group. This enrichment result together with the histopathological examination results (Figs. 2 and 3) underscore CYLD’s possible role of regulating neutrophil migration and activation. We then utilized gene set enrichment analysis (GSEA)to validate these findings. It was revealed that gene sets linked to chemokine signaling pathways, neutrophils degranulation and NF-κB response were all up-regulated in *Cyld*^*−/−*^ group (Fig. 4D-F).

**Fig. 5:**
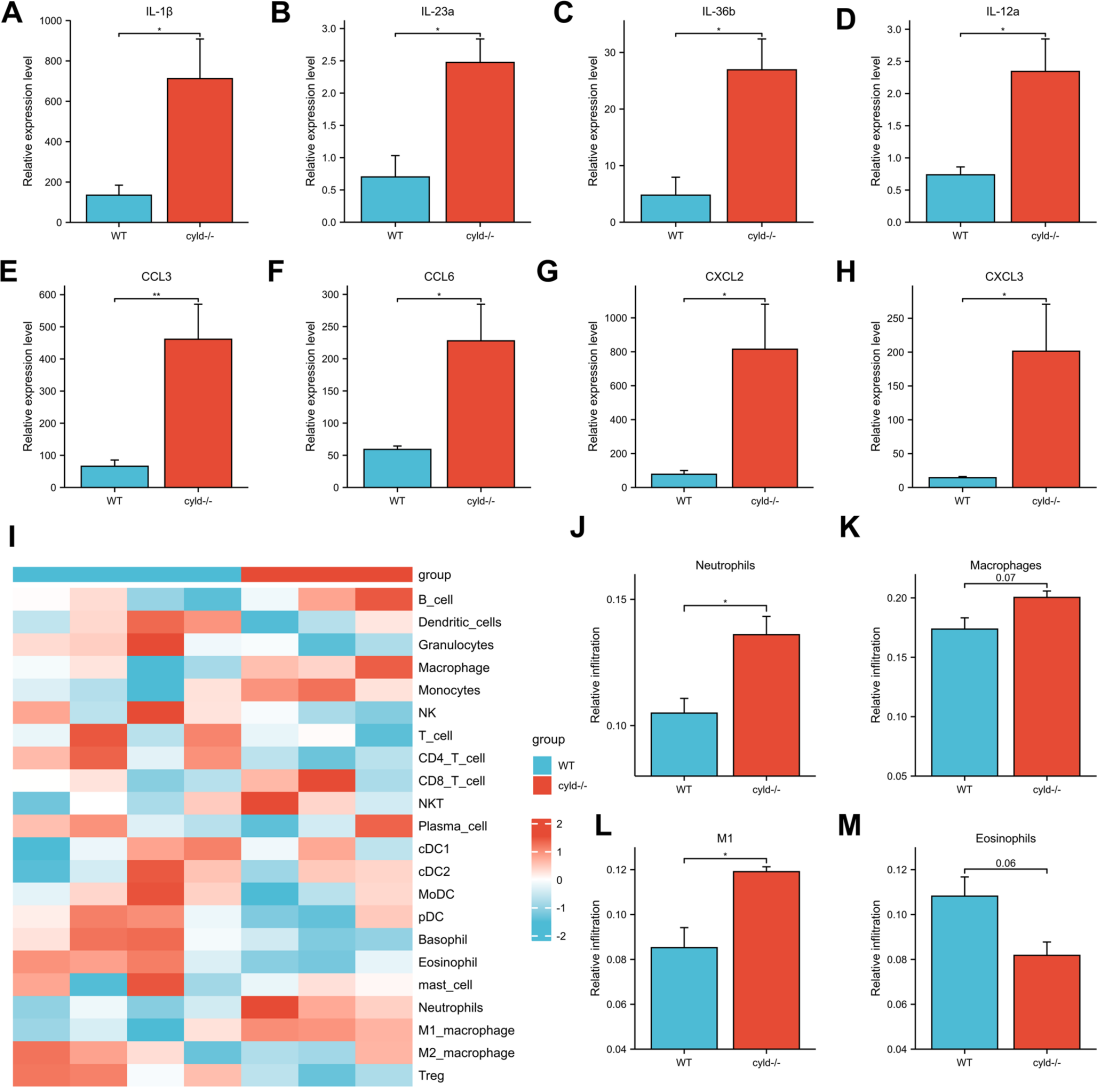
RNA-seq analysis reveals that *Cyld* knockout enhances the expression of psoriasis-related cytokines, neutrophil chemotaxis, and neutrophil infiltration in the IMQ model. **A** - **H** Expression of psoriasis-related cytokines (IL−1β, IL−22a, IL−36b, IL−12a) and neutrophil chemotaxis (CCL3, CCL6, CXCL2, CXCL3). **I** - **M** Immune cell infiltration was analyzed via CIBERSORT for immune cell profiling. Bar graphs show the relative abundance of key immune cells (Neutrophils, Macrophages, M1 macrophages, Eosinophils) in skin tissues. *p < 0.05.This experiment was repeated twice, with three mice in WT group and four mice in *cyld* ^*−/−*^ group

Next, we detected the mRNA expression levels of the genes encoding several key inflammatory cytokines and chemokines that are closely associated with psoriasis pathogenesis, including IL-1β, IL-23a, IL-36b, CCL3, CXCL2, CXCL3 and so on (Fig. 5A-H). The results showed a marked increase in the mRNA expression of these cytokines and chemokines in the *Cyld*^*−/−*^ group, further corroborating CYLD’s pivotal role in modulating inflammatory responses. Additionally, CIBERSORT analysis [24] showed a significant increase in neutrophil and M1 type macrophage populations in *Cyld*^*−/−*^ mice, consistent with the observed up-regulation of chemokine signaling and results above. These findings collectively suggest that CYLD deficiency exacerbates psoriatic inflammation by promoting cytokine production and immune cell infiltration. Notably, these data indicate that neutrophil infiltration and activation may both be increased in *Cyld*^*−/−*^ mice subsequent to IMQ treatment.

### CYLD Deficiency is Associated with Neutrophil Activation and NETs Formation in Human Psoriasis and a Mouse Model

**Fig. 6:**
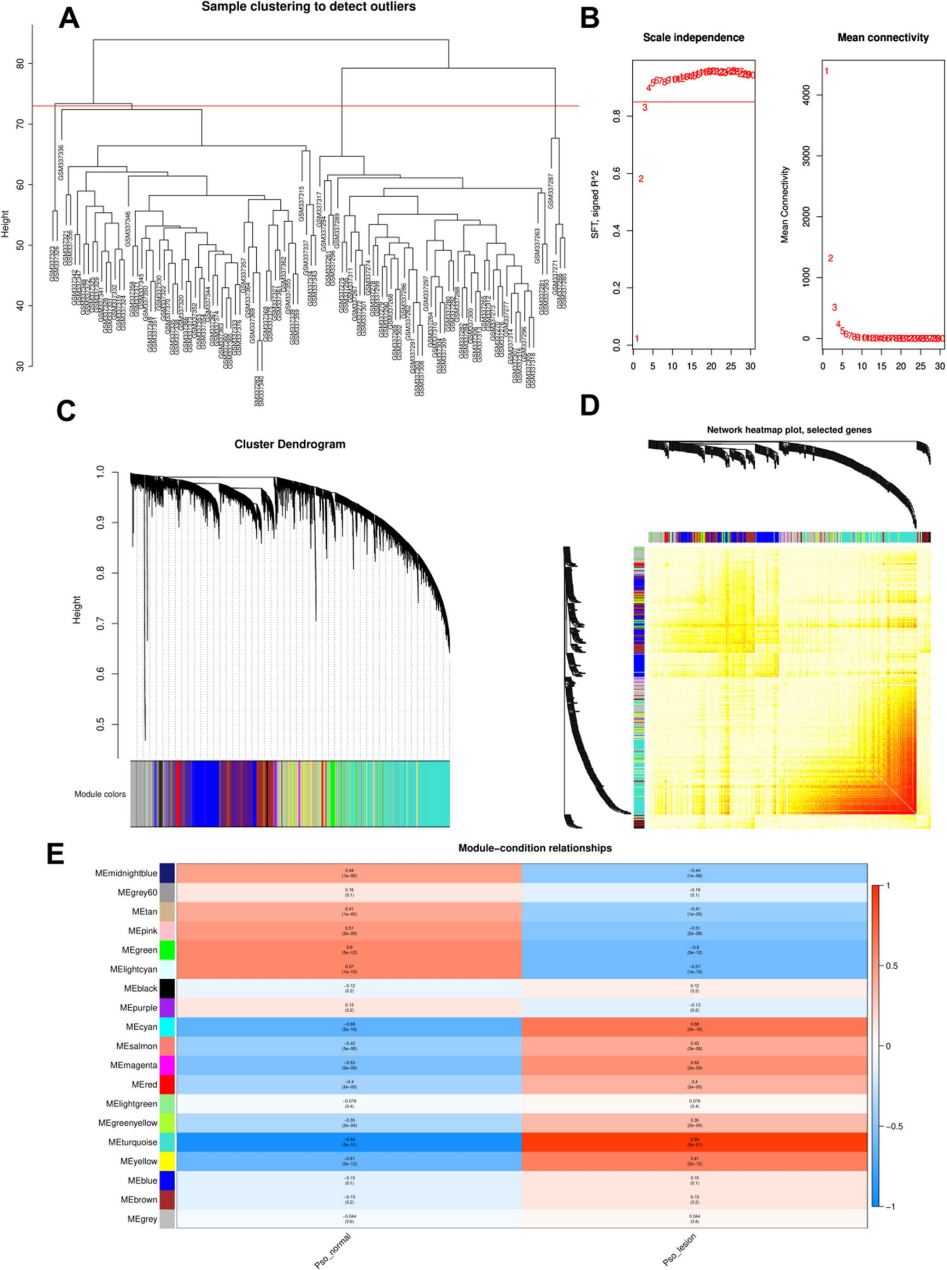
Weighted gene co-expression network analysis (WGCNA) of GSE13355. **A** Sample clustering to detect outliers. **B** Soft-thresholding power selection: The left panel (Scale Independence) and right panel (Mean Connectivity) show that a power value of 4 was chosen as the optimal parameter (balancing scale-free topology and connectivity). **C** The clustering dendrogram of genes. Genes are clustered into distinct modules (labeled by different colors at the bottom) based on co-expression patterns. **D** Gene co-expression network diagram. The heatmap (right) visualizes the co-expression relationships among genes, paired with the corresponding gene clustering dendrogram (left). **E** Gene dendrogram and gene co-expression network diagram. Rows represent co-expression modules, columns represent experimental conditions; color intensity (blue to red) and values indicate the correlation between each module and the condition (red = positive correlation, blue = negative correlation). This experiment was repeated twice

**Fig. 7:**
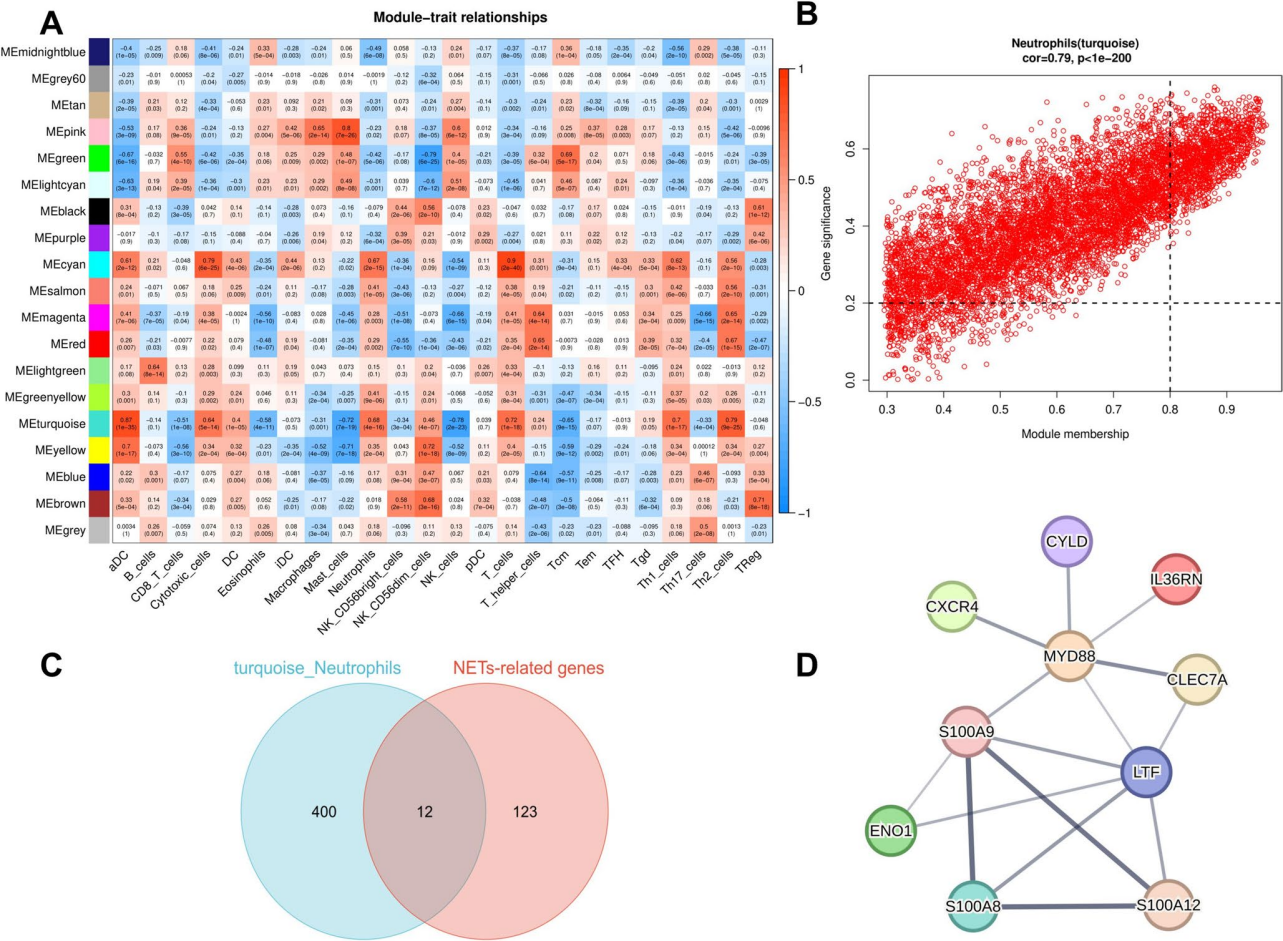
WGCNA analysis: Multi-component analysis of module - trait relationships and NETs - related gene networks. **A** Module-trait relationships heatmap. Rows represent co-expression modules. Columns denote different cell types (traits). Color intensity (blue to red) and values indicate the correlation between each module and trait (red = strong positive correlation, blue = strong negative correlation). **B** Scatter plot of gene significance vs. module membership (Neutrophils, turquoise module). The positive correlation (upward trend) implies that genes highly connected to the turquoise module are relevant to neutrophil traits. **C** Venn diagram of turquoise_Neutrophils and NETs-related genes. The turquoise circle (400 genes) = turquoise module-specific genes, the pink circle (123 genes) = NETs-related genes. **D** Protein-protein interaction (PPI) network of key genes and *CYLD*. Nodes represent proteins and edges represent interaction relationships, illustrating the connectivity between CYLD and these key molecules. This experiment was repeated twice

Neutrophil infiltration is a hallmark of psoriatic progression [7]. Their activation is often accompanied by the formation of neutrophil extracellular traps (NETs), which amplifies the early immune responses and contributes to the chronicity of the inflammation during psoriatic pathogenesis [9]. To explore the potential link between CYLD and NETs in human psoriasis, we performed Weighted Gene Co-expression Network Analysis (WGCNA) on the GSE13355 dataset. Sample clustering was used to identify and rule out the outlier samples (Fig. 6A). A soft threshold power of 4 was chosen, as it yielded a scale-free topology fitting index (R²) of 0.85 and an average connectivity approaching 0 (Fig. 6B), ensuring the network conformed to a scale-free distribution. Subsequently, we utilized CIBERSORT analysis to quantify the infiltration levels of various immune cells (such as neutrophils, Th1/Th17 cells, etc.) in skin samples from GSE13355 dataset and used these scores as phenotypic traits for WGCNA. Nineteen modules were generated via the dynamic tree cut algorithm (Fig. 7A). The MEturquoise module exhibited the highest correlation with both psoriasis (correlation coefficient = 0.94, *p* < 3e-51) and neutrophil infiltration (correlation coefficient = 0.68, *p* < 4e-16). It also had the highest module membership (kME) value (correlation coefficient = 0.79, *p* < 1e-200) (Fig. 7B). Therefore, the MEturquoise module was selected as the core gene set most significantly associated with both psoriasis progression and neutrophil infiltration. Genes within this module were further analyzed to identify hub genes involved in neutrophil infiltration by comparing them with a previously reported list of 136 NETs-related genes (NETRGs) [4, 25]. As shown in Figs. 7C and 12 candidate genes (SGK1, CLEC7A, CTSC, CXCR4, ENO1, F3, IL36RN, LTF, MYD88, S100A12, S100A8, and S100A9) were identified through the overlap between the 412 key module genes and NETRGs. A protein-protein interaction (PPI) network of these key genes (Fig. 7D) revealed that CYLD can interact intimately with MYD88, suggesting it is part of the regulatory network governing neutrophils during psoriasis.

**Fig. 8:**
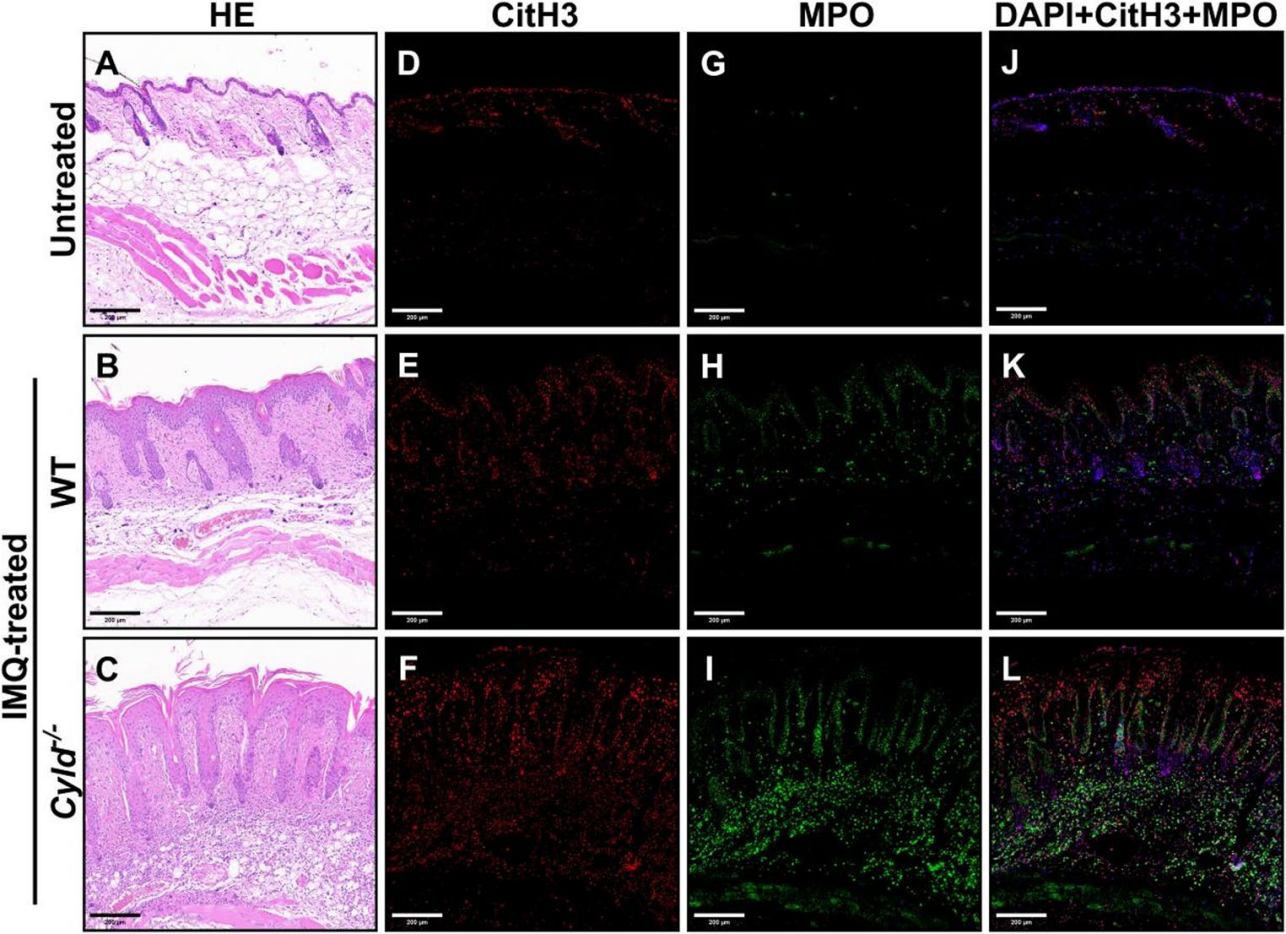
*Cyld*^*−/−*^ mice exhibit significantly enhanced NETs formation compared to WT mice in the IMQ model. Rows represent different treatment groups: “Untreated”, no specific treatment; “IMQ-treated WT”, WT mice treated with imiquimod; “IMQ-treated c*yld*^*−/−*^”, Cyld knockout mice treated with imiquimod. Colums represent different staining/labeling assays. **A**-**C**) HE staining; (**D**-**F**) Citrullinated histone H3 (CitH3) staining (red); (**G**-**I**) Myeloperoxidase (MPO) staining (green); (**J**-**L**) Merged images of citH3, MPO, and DAPI (nuclear stain, blue; right column). This experiment was repeated three times, with three mice in each group

To validate these findings in vivo, we assessed NETs formation in *Cyld*^*−/−*^ and WT mice post-IMQ treatment using immunofluorescence staining for DAPI, citrullinated Histone H3 (citH3), and Myeloperoxidase (MPO). In untreated mice, citH3 signals were detectable only in the epidermis, and MPO signals were almost absent (Fig. 8D-J). As expected, IMQ treatment significantly enhanced both signals, which were also evident in the dermis (Fig. 8E-K). In contrast, *Cyld*^*−/−*^ mice showed markedly increased levels of citH3 and MPO signals, with more extensive colocalization, indicating more pronounced NETs formation (Fig. 8F-L). These findings suggest that CYLD deficiency exacerbates neutrophil activation and NETs formation, thereby potentially contributing to psoriasis pathogenesis.

### The Effect of CYLD Deficiency on Neutrophil Infiltration May Act Through the Modulation of the NF-κB Pathway

**Fig. 9:**
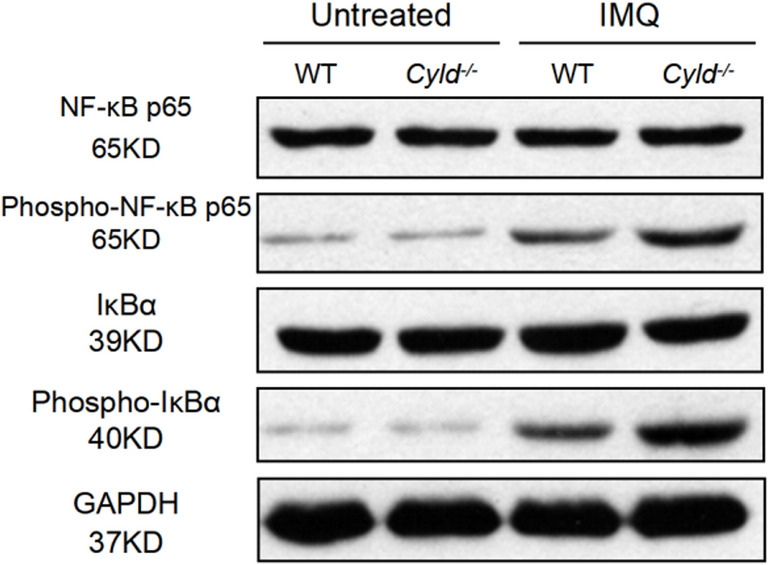
Western blot analysis of NF-κB signaling pathway components in WT and *Cyld*^*−/−*^ samples, with or without IMQ treatment. Protein lysates were probed for total NF-κB p65, phosphorylated (Phospho)-NF-κB p65, total IκBα, phosphorylated (Phospho)-IκBα, and GAPDH (loading control). This experiment was repeated three times, with three mice in each group

CYLD is a well-characterized negative regulator of the NF-κB pathway, which functions downstream of MyD88. Thus, we speculate that in the absence of CYLD, prolonged NF-κB activation promotes sustained neutrophil engagement, leading to excessive NETs production. To test this, we investigated NF-κB activation in the IMQ model. Compared with untreated controls, IMQ-treated WT mice exhibited increased phosphorylation of both P65 and IκBα (Fig. 9), indicating enhanced NF-κB activation. Importantly, *Cyld*^*−/−*^ mice showed significantly higher phosphorylation levels compared to WT mice (Fig. 9), suggesting that CYLD deficiency exacerbates NF-κB activation during IMQ-treatment. This result further supports our hypothesis that CYLD may play a crucial role in modulating NF-κB signaling, thereby influencing neutrophil activity and NETs formation in psoriatic conditions.

## Discussion

In this study, we demonstrate a significant association between psoriasis pathogenesis and CYLD expression, highlighting that CYLD-deficient mice present with aggravated psoriatic characteristics and increased inflammatory cell infiltration. Moreover, the depletion of CYLD facilitates elevated Ki67 expression and promotes neutrophil involvement within IMQ-induced psoriasis lesions, suggesting that CYLD deficiency accelerates neutrophil activation and infiltration.

The CYLD gene, initially identified for its role in skin tumors, has emerged as a critical regulator within the inflammatory and immune response pathways [26]. Given the known widespread roles of CYLD, it is unsurprising that it has been shown to play a role in a range of diseases, including tumors, inflammatory diseases and neurodegenerative disorders. CYLD’s role in skin pathology is particularly well-characterized. Notably, CYLD is a well-established tumor suppressor in inherited skin tumor syndromes collectively termed CYLD cutaneous syndrome (CCS), including cylindromas, spiradenomas, and trichoepitheliomas [27]. CCS patients develop multiple benign hair follicle-derived tumors on the head and torso, with nearly all tumors exhibiting loss of heterozygosity at the CYLD locus (chromosome 16q12-13), further abrogating CYLD function. Mechanistically, CYLD deficiency in CCS leads to unchecked activation of NF-κB signaling via accumulation of ubiquitinated substrates, including TRAF2, TRAF6 and BCL-3, thereby driving abnormal proliferation and survival of cutaneous epithelial cells [28, 29]. Beyond inherited syndromes, CYLD dysfunction contributes to sporadic skin cancers. In malignant melanoma, CYLD expression is downregulated by BRAF-mediated ERK (MAPK) activation via the transcription factor SNAL1, thereby releasing its inhibitory control over NF-κB and promoting tumor cell proliferation, invasion, and epithelial-mesenchymal transition [30]. CYLD also modulates skin inflammation by regulating key signaling pathways (e.g., NF-κB, Wnt, TGF-β) and cell death programs-both of which are central to skin immune homeostasis [31, 32]. Specifically, in the context of atopic dermatitis (AD), CYLD is highly expressed in macrophages of AD patients, and overexpressed CYLD inhibits the STAT1 and NF-κB pathways through K63-specific deubiquitination, thereby exacerbating AD-related inflammation and increasing infection risks [33].

Recently, bioinformatics research suggested the potential role of CYLD in the pathogenesis of psoriasis. The CYLD gene has been identified as one of the candidate genes involved in susceptibility to psoriasis, and two SNPs (rs4785452 and rs12925755) within it have been reported to be linked to psoriasis [18, 34]. In the 2009 study on French psoriasis families, these two SNPs were significantly associated with psoriasis susceptibility, with rs12925755’s minor allele A increasing disease risk and rs4785452 retaining significance in an enlarged sample set via FBAT analysis. Additionally, both SNPs showed enhanced association with psoriasis specifically in HLA-Cw6 carriers, indicating a synergistic interaction between CYLD variants and the major risk allele HLA-Cw6 [35]. Using clinical databases, we found that the expression of the CYLD gene shows significant increase either between psoriasis patients and healthy controls or between lesional and non-lesional skin of the same patient. This phenomenon was replicated in IMQ-induced psoriasis-like mouse models, where increased CYLD expression was recorded after day 4. Interestingly, we noticed that at the earlier time point, there was a marked reduction of CYLD. At this time point (Day2, Day3), the noticeable changes that we observed were the marked flushing on the dorsum (Fig. 2G), indicating an enhanced pro-inflammatory response accompanied with the transient down-regulation of CYLD. This may imply a possible association between CYLD downregulation and initial inflammation at the lesion site, which, in most cases, is induced by innate immune cells, such as neutrophils, macrophages or dendritic cells. However, how the expression of CYLD is regulated requires further research to clarify.

Consistent with the role of CYLD in other inflammatory contexts, such as inflammatory bowel disease [36], we first demonstrate that CYLD also exerts an inhibitory effect on inflammatory responses in psoriasis. Interestingly, pathological data, RNA-seq analysis, and NETs immunofluorescence staining all point to a negative correlation between CYLD expression and neutrophil infiltration and activation during IMQ-treatment. However, at this time point (Day 5), there was no significant increase in F4/80^+^ and CD3^+^ cells in the skin lesions of CYLD-deficient mice compared to control groups (Fig. S1), suggesting that the absence of CYLD might mainly affect infiltration of neutrophils rather than macrophages or T cells. RNA-seq analysis also supported this notion, as pathway enrichment analysis reveals marked activation of neutrophil-related pathways and CIBERSORT immune cell profiling analysis only shows a significant increase in the abundance of neutrophils but not other types of innate immune cells in the skin of CYLD-deficient mice. However, the underlying mechanism by which CYLD regulates neutrophil infiltration remains unclear. As mentioned above, the well-known mechanism of CYLD in regulating cutaneous tumors and inflammatory diseases lies in its role as a deubiquitinating enzyme that negatively regulates NF-κB-related signaling pathways [27–33]. In our IMQ model, CYLD deficiency indeed induces increased activation of NF-κB. This enhanced NF-κB activation may contribute to the elevated expression of neutrophil-recruiting chemokines, such as CCL3 and CXCL2 (Fig. 5), in the skin of CYLD-deficient mice. Some tumor-related studies have found that NF-κB may act as a “power pump” in neutrophil-related inflammatory positive feedback loops [37–39]. This is a possible mechanism by which psoriasis may maintain its chronic inflammatory state as some recent studies have shown that sustained NF-κB activity in keratinocytes can drive the continuous production of pro-inflammatory cytokines and chemokines, thereby perpetuating neutrophil infiltration and tissue inflammation [40–42]. Besides, NET formation is also believed to be closely associated with the sustained inflammatory response in psoriasis [8–10]. Pro-inflammatory activity of NETs is dependent upon activation of TLR4/IL-36R crosstalk and MyD88/NF-κB downstream signaling [8]. Herein, we also observed elevated levels of NET markers, such as Cit-H3 and MPO, in *Cyld*^*−/−*^ mice. Another possible mechanism by which CYLD may regulate the immune response in the IMQ model is directly through influencing the TLR4 signaling, as it has been demonstrated that CYLD acts as a negative regulator of the TRAF6 complex in the TLR4 signaling pathway [43]. Further in-depth investigations into the mechanism by which CYLD regulates neutrophils are required.

These findings suggest that targeting CYLD function or expression may inhibit inflammatory responses during psoriasis pathogenesis. For instance, phosphorylation of CYLD at Ser568 specifically enhances its deubiquitinating activity, enabling it to act as a highly active Lys63-linked ubiquitin (Lys63-Ub) deubiquitinase. In this activated state, CYLD suppresses excessive NF-κB activation by cleaving Lys63-Ub chains in receptor signaling complexes. Another key site is Asp215 (D215) of CYLD, which is cleaved by Caspase-8. Mutation of D215 abrogates CYLD cleavage, thereby preserving its full-length form and enhancing its deubiquitinating activity—ultimately conferring a more pronounced inhibitory effect on NF-κB signaling. These sites of CYLD may serve as potential entry points for future investigations into the relationship between CYLD and psoriasis pathogenesis, as well as the development of novel therapeutic interventions for psoriasis. While we are enthusiastic about these targets, we should exercise caution regarding the potential pleiotropic effects of activating potential mutations at these key sites on cutaneous inflammatory pathways—a matter that warrants further investigation.

Collectively, our findings indicate that CYLD modulates NETs and likely attenuates IMQ-induced skin inflammation by regulating neutrophil function. Further studies to elucidate the precise mechanisms by which CYLD regulates psoriasis pathogenesis are needed to develop targeted therapeutic approaches for this disease.

## Methods

### Mice

*Cyld* ^*−/−*^ mice were generously gifted by Professor Haibing Zhang (Shanghai Institutes for Biological Sciences, China) initially. *Cyld*^*+/−*^ mice were generated by a CRISPR-Cas9 approach. *Cyld*^*−/−*^ mice were generated by intercrossing *Cyld*^*+/−*^ males and females, and their genotyping was performed as described previously [44]. (Genotying primers used in this study: CYLD-F: 5′-GGGACTTACAGCGAGTTCAT-3′ and CYLD-R: 5′-ATAGATCAGTGGTAGAGGGT − 3′). C*yld*^*−/−*^ mice (C57BL/6 background) and wild-type (WT) C57BL/6 mice (Keyu Animal Breeding Center, Beijing, China) between 6 and 10 weeks of age were used in accordance with local institutional guidelines on animal experiments, regular hygiene monitoring, and specific locally approved protocols compliant with the National Institutes of Health Guide for the Care and Use of Laboratory Animals and relevant ethical regulations. The animal study was reviewed and approved by the Institutional Animal Care and Use Committee of Chinese PLA General Hospital.

### Imiquimod Model of Psoriatic Skin Inflammation

Dorsal hair of female mice at 8–12 weeks of age were removed with a commercially available electric hair remover. The hair removed mice were monitored for another two days then randomly treated with 62.5 mg IMQ cream (5%, Mingxin Pharmaceuticals, Sichuan, China) and placebo cream daily for six consecutive days. The severity scoring on erythema, induration, and desquamation was conducted every day as previously described [42]. The score is classified as five levels from 0 to 4, which 0 is the lowest indicating no severity and 4 is the highest severity.

### Western Blotting

Tissues were lysed in RIPA lysis buffer containing 50 mM Tris (pH = 8.0), 1 mM EDTA, 150 mM NaCl, 0.1% SDS, 1% Triton X-100, 0.2% sodium deoxycholate, 50 mM NaF, 1×proteinase inhibitor cocktail (Abcam, ab65621), 2 mM PMSF, and 5 mM NEM. The lysates were cleared by centrifugation for 30 min at 12,000×g quantified by BCA kit (P0010S, Beyotime), and then mixed with SDS sample buffer and boiled at 96 °C for 10 min. The samples were separated using SDS-PAGE, transferred to PVDF membrane (Millipore) with 110 v for 2 h. The proteins were detected by using a chemiluminescent substrate (Thermo Scientific, 34577). Normalized densitiomentry was calculated by ImageJ software (1.54 g, National Institutes of Health, USA). The following antibodies were used for western blotting: CYLD (1:2000, Cell Signaling Technology, 8462 S), IkBα (1:2000, Cell Signaling Technology, 9242 S), p-IkBα (1:1000, Cell Signaling Technology, 9246 S), p-P65 (1:2000, Cell Signaling Technology, 3033 S), P65 (1:2000, Cell Signaling Technology,4764 S), P65(1:2000, Cell Signaling Technology, 8242 S), GAPDH (1:20,000, Abcam, ab8245).

### Histology and Immunohistochemical Staining

Tissue was fixed overnight with 10% formaldehyde solution (Sigma, HT501128). After dehydration and paraffin embedding, 4 μm sections were prepared and stained with hematoxylin & eosin. Antigen retrieval was conducted by immersing slides in citrate buffer. Prior to staining, endogenous peroxidase was inhibited by 3% H_2_O_2_, and non-specific protein binding was blocked by incubating the slides in 1% bovine serum albumin. The slides were then incubated with primary antibody at 4 °C overnight, followed by biotinylated secondary antibody for 30 min at room temperature. Images were acquired using an Pannoramic MIDI digital camera system (3DHISTECH, Hungary) and SlideViewer (2.8.0.216379, 3DHISTECH, Hungary). Normalized densitiomentry was calculated by ImageJ software (1.54 g, National Institutes of Health, USA).

Immunohistochemical staining was assessed by two independent investigators. Primary antibodies used in this study: Ki67 (Cell signaling technology, 9027), Ly6G (Cell signaling technology, S7048T), CD3 (Abcam, ab16669), F4/80(Abcam, ab111101).

### RNA-seq analysis 

For the RNA-seq analysis, three *Cyld*^*−/−*^ mice and four WT mice were subjected to the IMQ-induced model as described above. On day 5, the mice were anesthetized, and lesional skin tissues were excised. After rinsing with normal saline and removing subcutaneous tissues, total RNA was extracted. Then the sequencing was performed by Biomarker Technologies Co., Ltd (Beijing, China). DEGs between the *Cyld*^*−/−*^ and WT group were selected by the limma package (v3.52.2) with adjusted P value < 0.05 and |log_2_FC|>2. The volcano plot was illustrated by the ggplot2 package (v3.4.4). The heatmap was illustrated by the R package ComplexHeatmap (v2.13.1) [45]. KEGG/GO and GSEA analyses were performed using the R package clusterProfiler (v4.4.4) and illustrated by the ggplot2 (v3.4.4). All the analyses were performed using the R software (v4.2.1), developed by the R Core Team (R Foundation for Statistical Computing, Vienna, Austria).

### WGCNA and Filtering for Key Module Genes

The co-expression network was constructed by WGCNA, which was performed using the OmicStudio tools at https://www.omicstudio.cn/tool/WGCNA (v1.71, R version 4.1.3, ggplot2 package 3.3.6). All samples were from GSE13355 data sets (64 healthy vs. 58 psoriatic samples). Briefly, the samples were clustered and outliers were removed (GSM337266, GSM337271, GSM337285, GSM337287, GSM337323, GSM337326) to ensure the accuracy of the analysis. Network topology analysis ensured a scale-free topology network with the defined soft-thresholding power of 4. A total of 19 modules were identified based on the dynamic tree cutting algorithm with the parameters of minModuleSize at 30 and mergeCutHeight at 0.25. The cluster dendrogram was obtained by calculating adjacency and similarity. The modules were partitioned by dynamic tree cutting algorithm. The MEDissThres was then set to 0.5 to merge similar modules. Next, we evaluated the correlation between each module and both PSO and neutrophil infiltration. Finally, the genes in the key module with |Gene significance|> 0.2, *P* value ≤ 0.05, and |Module memebership|> 0.8 were identified as key module genes for follow-up analysis.The 136 neutrophil extracellular traps-related genes (NETRGs) (Supplementary Table 1) were obtained from previous report [25]. A PPI network was created on the basis of candidate genes via the STRING database.

### CIBERSORT for Immune Cell Profiling

CIBERSORT analysis was performed according to the methods previously reported [46, 47], using the OmicStudio tools at https://www.omicstudio.cn/tool.

### Immunofluorescence

Paraffin sections were deparaffinized in xylene, followed by rehydration in absolute ethanol and thorough rinsing with distilled water. Antigen retrieval was performed with continuous monitoring to prevent buffer evaporation and section desiccation. Sections were then washed with PBS for three times. Non-specific binding sites were blocked by covering tissue sections with 3% BSA (10% donkey serum was used for goat-derived primary antibodies) and incubated at room temperature for 30 min. Primary antibodies were added, and sections were flat-mounted in a humidified chamber, followed by incubation overnight at 4 °C. After washing with PBS, sections were incubated with secondary antibodies at room temperature for 50 min in the dark. For nuclear counterstaining, sections were washed with PBS, incubated with DAPI solution for 10 min at room temperature in the dark, and then rinsed briefly. Tissue autofluorescence was quenched by sequential washing with PBS, incubation with Autofluorescence Quencher Solution B for 5 min, and rinsing under running water. Finally, sections were mounted with anti-fade mounting medium and coverslipper. Images were acquired using an ortho-fluorescent microscopy (NIKON ECLIPSE C1, (Nikon, Japan) and a Pannoramic MIDI digital imaging system (3DHISTECH, Hungary). The following antibodies were used for immunofluorescence: Anti-Histone H3 (citruline R2 + R8 + R17, Abcam, ab5103), Goat Anti-Rabbit IgG Alexa Fluor 488 (1:400, Servicebio, GB25303, Wuhan, China), Goat Anti-Rabbit IgG CY3 (1:300, Servicebio, GB21303, Wuhan, China), DAPI (Abcam, ab104139).

### Statistical Analysis

All bioinformatics analyses were carried out in R language. All data were presented as mean ± standard deviation (s.d.), and the student’s t-test was used to analyze the difference between the two groups. Correlations among data were analyzed using Spearman’s correlation analysis. The software used was SPSS version 27.0 and GraphPad Prism version 8.0. *P* < 0.05 was considered statistically significant. 

## Supplementary Information

Below is the link to the electronic supplementary material.

Supplementary Material 1

Supplementary Material 2

Supplementary Material 3

## Data Availability

No datasets were generated or analysed during the current study.
