# Supplementary material for: CYLD Limits Neutrophil-Driven Psoriatic Inflammation
Source: Inflammation. 2026 Jan 20;49(1):57. doi: 10.1007/s10753-026-02452-3 (PMC12883520; doi:10.1007/s10753-026-02452-3)
Supplement: Supplementary file 1 — Supplementary Material 1 [file 10753_2026_2452_MOESM1_ESM.docx]

**CYLD Limits Neutrophil-Driven Psoriatic Inflammation**

Zhenzong Fa^1^, Zeping Huang^2^, Yi Shang^3^, Yang Yang^1^, Qun Xie^4^, Runping Yang^1^.

1 Department of Dermatology, the Sixth Medical Center, Chinese PLA General Hospital, 100048, Beijing, China

2 Outpatient Department, the Sixth Medical Center, Chinese PLA General Hospital, 100048, Beijing, China

3 Outpatient Department, Chinese PLA General Hospital, 100048, Beijing, China

4 Department of Anesthesiology, the Fourth Medical Center, Chinese PLA General Hospital, 100048, Beijing, China

**Supplementary material**

Table. S1 The list of 136 NETRGs[20].

| SGK1 | CCDC25 | CXCL2 | HIF1A | ITGB2 | MIR21 | PARVB | SOCS3 |
| --- | --- | --- | --- | --- | --- | --- | --- |
| ACTB | CCL2 | CXCR4 | HMGB1 | KCNN3 | MIR223 | PF4 | SPP1 |
| ACTG1 | CCL3 | CYBB | HRG | KLF2 | MMP9 | PIK3CA | SRC |
| ACTN1 | CCL4 | DEFA3 | IL12A | KRT10 | MNDA | PKM | STAT3 |
| ACTN4 | CCL5 | DNAJB1 | IL17A | LCP1 | MPO | PROCR | SUCNR1 |
| AKT1 | CD177 | DNASE1 | IL1B | LDLR | MTOR | PRTN3 | SYK |
| AKT2 | CD274 | ELANE | IL1RL1 | LPAR3 | MYD88 | PTAFR | TICAM1 |
| ARPIN | CD44 | ENO1 | IL33 | LTF | MYH9 | RIPK1 | TIMP1 |
| ATG7 | CEBPB | ENTPD4 | IL36RN | LYZ | NFE2L2 | RIPK3 | TKT |
| AZU1 | CFTR | F2RL2 | IL5 | MAPK1 | NFIL3 | S100A12 | TLR2 |
| C3 | CLEC4E | F3 | IL6 | MAPK14 | NFKBIA | S100A8 | TLR4 |
| C3AR1 | CLEC6A | FCAR | IL8 | MAPK3 | NLRP3 | S100A9 | TLR7 |
| C5AR1 | CLEC7A | FCGR2B | ILK | MAPK7 | NOX4 | S1PR2 | TLR8 |
| CAMP | CSF3 | FGL2 | IRAK4 | MCOLN3 | OPA1 | SELP | TLR9 |
| CARD11 | CTSC | GPBAR1 | IRF1 | MFN1 | ORAI1 | SELPLG | TNF |
| CASP1 | CTSG | GSDMD | ITGAM | MFN2 | P2RX1 | SGK1 | TNFAIP3 |
| CAT | CXCL1 | H2AX | ITGB1 | MIR146A | PADI4 | SIGLEC14 | WASL |


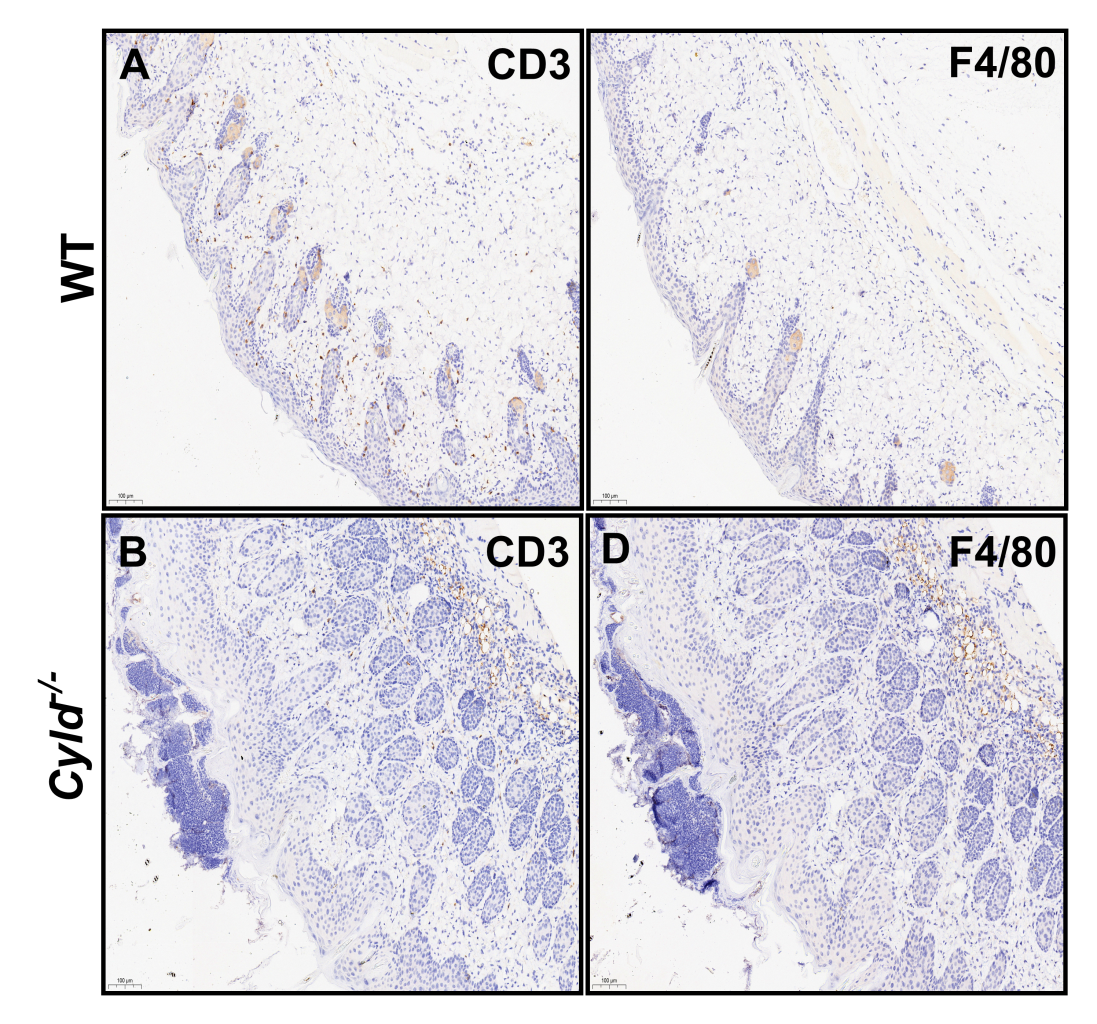


Fig.S1 *Cyld* depletion had no effect on CD3+ or F4/80+ cells in IMQ-induced psoriasis lesions.

Immunohistochemical analysis of CD3 (A, B) and F4/80 (C, D).
