# Supplementary material for: CYLD Limits Neutrophil-Driven Psoriatic Inflammation
Source: Inflammation. 2026 Jan 20;49(1):57. doi: 10.1007/s10753-026-02452-3 (PMC12883520; doi:10.1007/s10753-026-02452-3)
Supplement: Supplementary file 2 — Supplementary Material 2 [file 10753_2026_2452_MOESM2_ESM.docx]

Original images of Fig1.D

CYLD

Day4

Day6

Day0

Day2

Untreated

IMQ


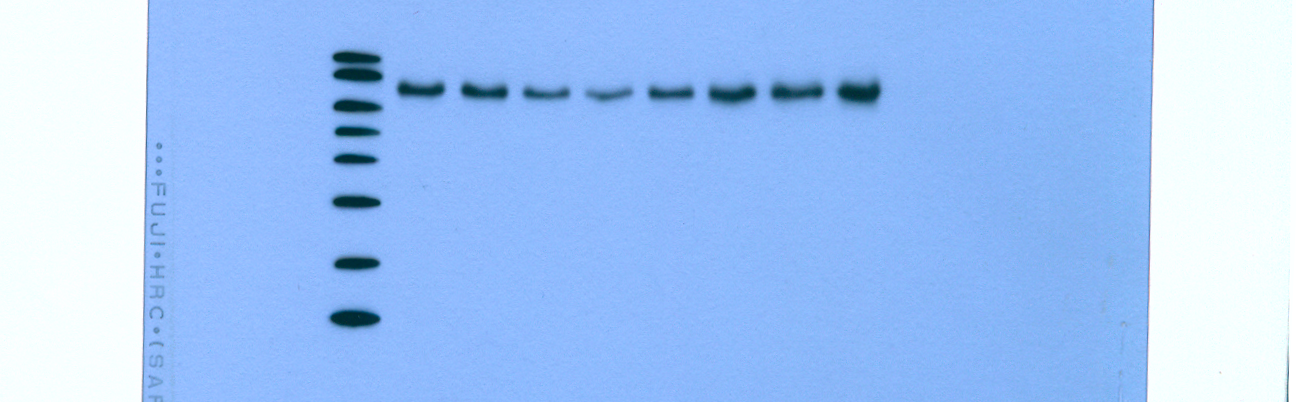


108KD

GAPDH


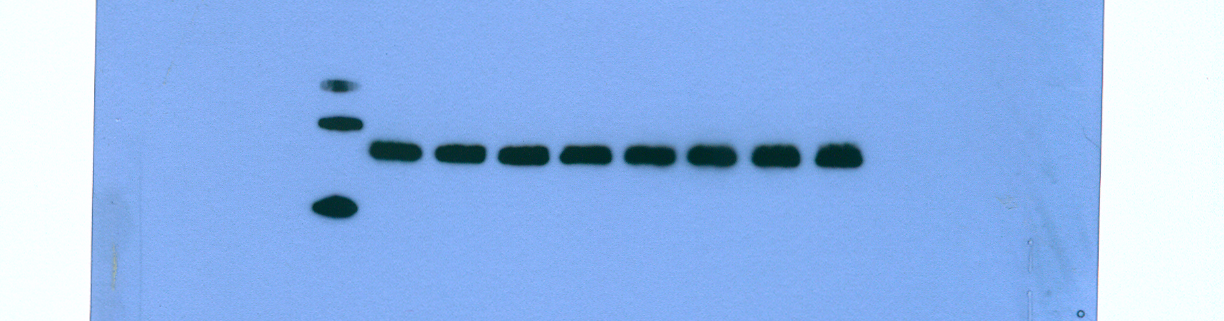


Day4

Day6

Day0

Day2

Untreated

IMQ

37KD
