# Supplementary material for: CYLD Limits Neutrophil-Driven Psoriatic Inflammation
Source: Inflammation. 2026 Jan 20;49(1):57. doi: 10.1007/s10753-026-02452-3 (PMC12883520; doi:10.1007/s10753-026-02452-3)
Supplement: Supplementary file 3 — Supplementary Material 3 [file 10753_2026_2452_MOESM3_ESM.docx]

Fig.S2 Original images of Fig.9

P65


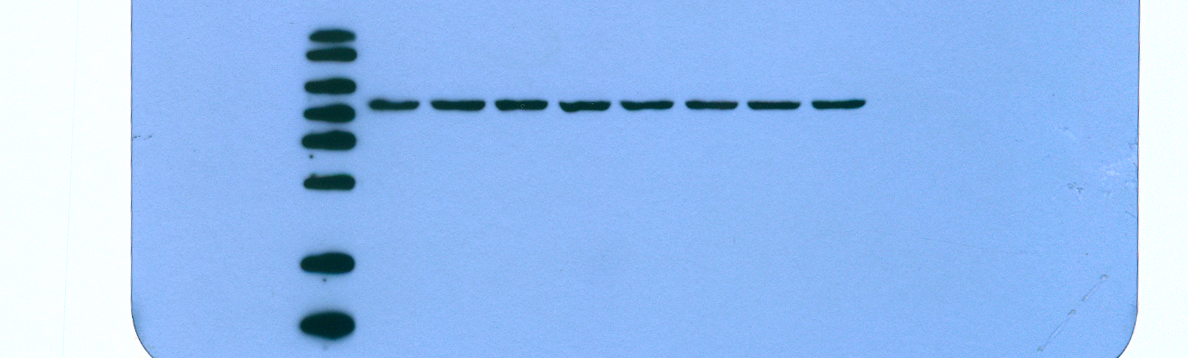


65KD

Untreated

WT

*Cyld^-/-^*

WT

*Cyld^-/-^*

IMQ

Phospho-P65


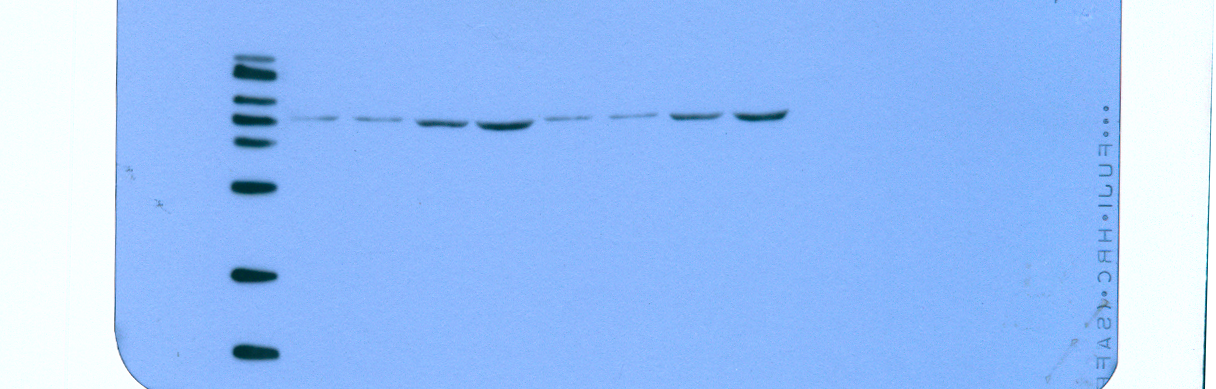


65KD

Untreated

WT

*Cyld^-/-^*

WT

*Cyld^-/-^*

IMQ

IκBα
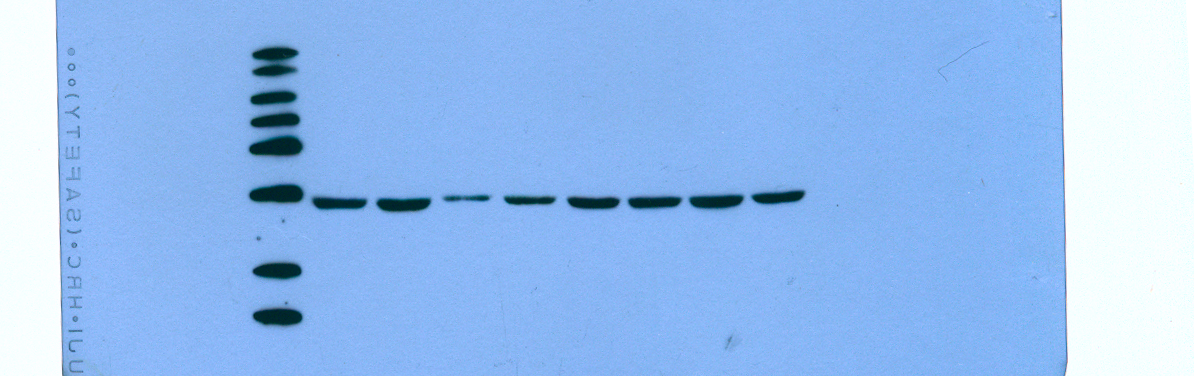


39KD

Untreated

WT

*Cyld^-/-^*

WT

*Cyld^-/-^*

IMQ

Phospho-IκBα


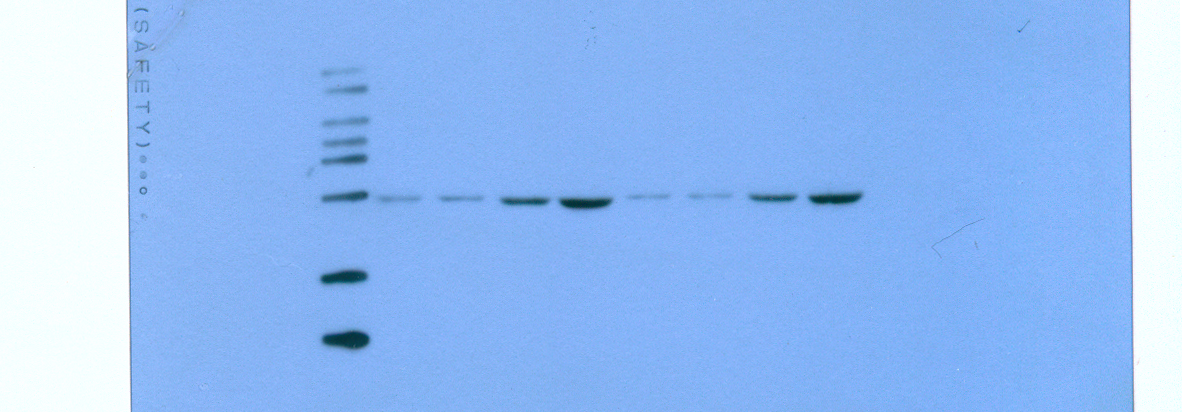


40KD

Untreated

WT

*Cyld^-/-^*

WT

*Cyld^-/-^*

IMQ

GAPDH


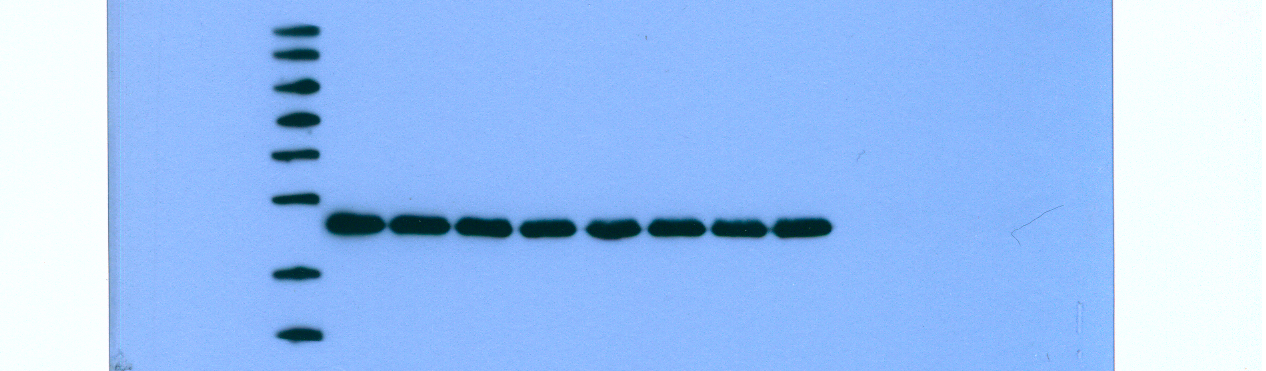


Untreated

WT

*Cyld^-/-^*

WT

*Cyld^-/-^*

IMQ

37KD
